# Effect of First Trough Vancomycin Concentration on the Occurrence of AKI in Critically Ill Patients: A Retrospective Study of the MIMIC-IV Database

**DOI:** 10.3389/fmed.2022.879861

**Published:** 2022-04-14

**Authors:** Longzhu Li, Luming Zhang, Shaojin Li, Fengshuo Xu, Li Li, Shuna Li, Jun Lyu, Haiyan Yin

**Affiliations:** ^1^Department of Intensive Care Unit, The First Affiliated Hospital of Jinan University, Guangzhou, China; ^2^Department of Clinical Research, The First Affiliated Hospital of Jinan University, Guangzhou, China; ^3^Department of Orthopaedics, The First Affiliated Hospital of Jinan University, Guangzhou, China; ^4^School of Public Health, Xi'an Jiaotong University Health Science Center, Xi'an, China; ^5^Guangdong Provincial Key Laboratory of Traditional Chinese Medicine Informatization, Guangzhou, China

**Keywords:** trough concentration of vancomycin, critically ill, AKI, mortality, nephrotoxicity

## Abstract

**Background:**

Vancomycin can effectively inhibit Gram-positive cocci and is widely used in critically ill patients. This study utilized a large public database to explore the effect of patients' first vancomycin trough concentration (FVTC) on the occurrence of acute kidney injury (AKI) and mortality after receiving vancomycin treatment in intensive care unit (ICU).

**Methods:**

Critically ill patients who used vancomycin in the Medical Information Mart for Intensive Care (MIMIC) IV have been retrospectively studied. The outcomes included the occurrence of AKI during the use of vancomycin or within 72 h of withdrawal, ICU mortality and hospital mortality. Restricted cubic splines (RCS) were used to analyze the linear relationship between FVTC and the outcomes. Multivariate logistic/Cox regression analysis was used to analyze the association between patient's FVTC and the occurrence of AKI, ICU mortality, and in-hospital mortality.

**Results:**

The study ultimately included 3,917 patients from the MIMIC-IV database who had been treated with vancomycin for more than 48 h. First of all, the RCS proved the linear relationship between FVTC and the outcomes. After controlling for all covariates as confounders in logistic/Cox regression, FVTC was a risk factor with the occurrence of AKI (OR: 1.02; 95% CI: 1.01–1.04), ICU mortality (HR: 1.02; 95% CI: 1.01–1.03), and in-hospital mortality (HR: 1.02; 95% CI: 1.01–1.03). Moreover, patients were divided into four groups in the light of the FVTC value: group1 ≤ 10 mg/L, 10 <group 2 ≤ 15 mg/L, 15 <group 3 ≤ 20 mg/L, group4 > 20 mg/L. Categorical variables indicated that group 3 and group 4 had a significant relationship on the occurrence of AKI [group 3: (OR: 1.36; 95% CI: 1.02–1.81); group 4: (OR: 1.76; 95% CI: 1.32–2.35)] and ICU mortality [group 3: (HR: 1.47; 95% CI: 1.03–2.09); group 4: (HR: 1.87; 95% CI: 1.33–2.62)], compared to group 1, while group 4 had a significant effect on in-hospital mortality (HR: 1.48; 95% CI: 1.15–1.91).

**Conclusions:**

FVTC is associated with the occurrence of AKI and increased ICU and in-hospital mortality in critically ill patients. Therefore, in clinical practice, patients in intensive care settings receiving vancomycin should be closely monitored for FVTC to prevent drug-related nephrotoxicity and reduce patient mortality.

## Introduction

Vancomycin is a glycopeptide antibiotic that has inhibitory effects on *Staphylococcus* (including methicillin-resistant *Staphylococcus aureus*, MRSA), *Streptococcus pneumoniae, Streptococcus pyogenes*, etc. (1, 2); it is the first-line drug for the treatment of MRSA (3, 4). However, the treatment window of vancomycin is narrow, and its effects during clinical treatment vary among different individuals, so vancomycin can often lead to adverse effects along with antibacterial therapy. As such, therapeutic drug monitoring (TDM) is required. As a time-dependent drug, its clinical efficacy measured by pharmacokinetic/pharmacodynamic parameter (PK/PD), which is 24 h drug time at area under concentration–time curve/minimal inhibitory concentration (AUC/MIC) (5). Although the most recent 2020 Infectious Diseases Society of America recommends that vancomycin should not be administered based on trough concentrations, but rather advocates AUC/MIC-guided dosing to achieve clinical efficacy (6). But there is still a gap between recommendations and practice, as estimating AUC/MIC in the clinical setting is difficult (7). Monitoring vancomycin trough concentration (VTC) prior to the fourth dose is often used as a simpler proxy to provide guidance on the appropriate use of vancomycin (8, 9).

Similar to many other drugs, vancomycin is excreted from the body mainly through the filtration of the kidney glomeruli and the secretion of the renal tubules. Acute kidney injury (AKI) is the main serious adverse drug reaction, which may affect up to 40% of patients treated with vancomycin. Moreover, it was demonstrated that when VTC was maintained at 15–20 mg/L in patients treated with vancomycin, it to be connected with a high incidence of AKI (10).Vancomycin-related AKI is associated with increased length of hospital stay, high cost, and high mortality (11). The clinical significance of VTC monitoring has been proven (12), and it is considered an accurate and practical indicator that reflects the effectiveness of medication and reduces nephrotoxicity (13, 14). A multicenter, prospective study revealed increased risk of nephrotoxicity in patients with MRSA infection when VTC is above 15 mg/L (15). Another prospective multicenter study recently reported by Liang et al. supported the association of VTC with nephrotoxicity in patients with Gram-positive bacterial infection, but not statistically associated with clinical outcomes (16). Vancomycin is widely used in intensive care units (ICU) mainly for bloodstream infections, meningitis, and infective endocarditis caused by Gram-positive bacteria (17). The special disease state of critically ill patients often leads to changes in the PK/PD of vancomycin in the body, besides, the combined use of multiple drugs, the coexistence of multiple underlying diseases and organ dysfunction may lead to reduced renal function in these patients (18, 19). Therefore, scholars should conduct in-depth study on the connection between VTC and critically ill patients. This study focused on ICU patients to investigate the effect of the first VTC (FVTC) after vancomycin treatment on the incidence of AKI and mortality in critically ill patients.

## Methods

### Data Source

This research was performed on a large, free, public database, namely, Medical Information Mart for Intensive Care (MIMIC) IV (20, 21). MIMIC is funded by multiple medical doctors and computer scientists from the Beth Israel Deaconess Medical Center (BIDMC), Massachusetts Institute of Technology, Massachusetts General Hospital, and Oxford University under the guidance of the National Institutes of Health and jointly established in 2003 (22). The latest version, MIMIC-IV 1.0, was updated in March 2021 and contains comprehensive information on ICU patients at BIDMC from 2008 to 2019 (23). All personal information about patients in the database is hidden, so informed consent is not necessary. The author of this study completed relevant courses and obtained a certificate to access the database (certificate number 38601114).

### Population Selection Criteria

**Figure 1:**
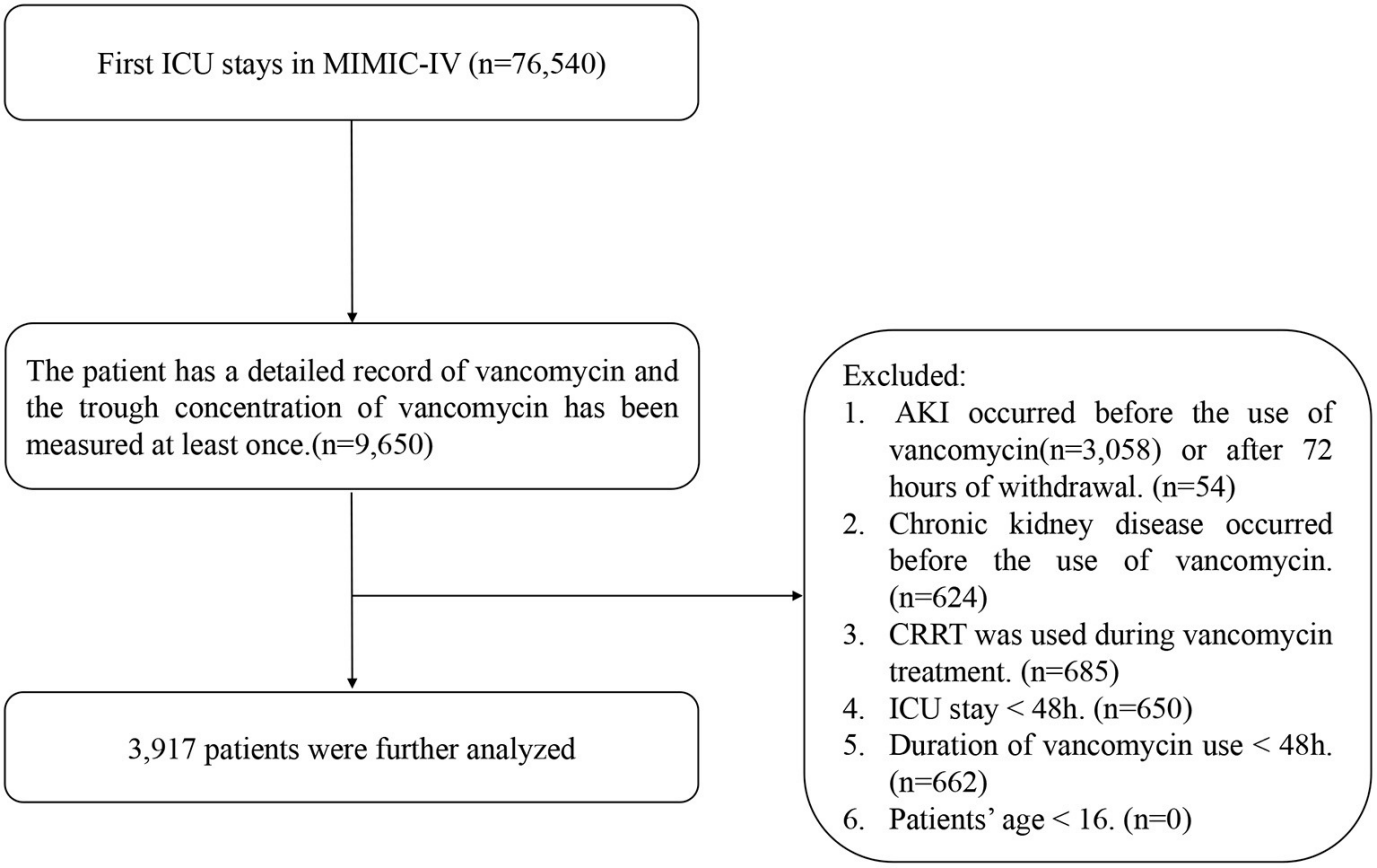
Flowchart of study population selection.

The inclusion criteria included the following: patients admitted to the ICU for the first time, use of vancomycin in the ICU, detailed record of start and end time, and VTC was measured at least once. The exclusion criteria were as follows. (1) AKI occurred before the use of vancomycin or after 72 h of withdrawal (24), as indicated by changes in serum creatinine or urine output as defined by KDIGO (25): an acute increase in the absolute level of serum creatinine of more than 0.3 mg/dL; a change of 50% higher in the serum creatinine level from baseline within a 48 h period; or decreased glomerular filtration rate to <0.5 mL/kg/ h for more than 6 h. (2) Chronic kidney disease occurred before the use of vancomycin. (3) CRRT was used during vancomycin treatment. (4) The patient's ICU stay was <48 h. (5) Duration of vancomycin use less than 48 h. (6) Patients younger than 16 years old (Figure 1).

### Outcomes and Covariates

The primary outcome was the occurrence of AKI during the use of vancomycin or within 72 h of withdrawal (24). The secondary outcomes included ICU mortality and hospital mortality.

Missing data in public databases are very common. The missing values of the data included in this study were all below 25%. The structured query language was used for data extraction. The information included age, weight, gender, SOFA score, type of patient's first admission to ICU, length of stay in ICU and in hospital, ventilator use, vasopressor use, and comorbidities, including sepsis, congestive heart failure, hypertension, diabetes, cancer, chronic obstructive pulmonary disease (COPD), liver disease, and stroke. Source of infection, infectious pathogen, co-infection with Gram-negative bacilli, duration of vancomycin use. Vital signs first monitored within 24 h of entering ICU were heart rate, mean arterial pressure, temperature, and respiratory rate. The first laboratory test results after entering ICU consisted of white blood cell (WBC), hemoglobin, platelet, potassium, sodium, chloride, bicarbonate, albumin, creatinine, urea nitrogen (BUN), and glucose.

### Statistical Analyses

**Figure 2:**
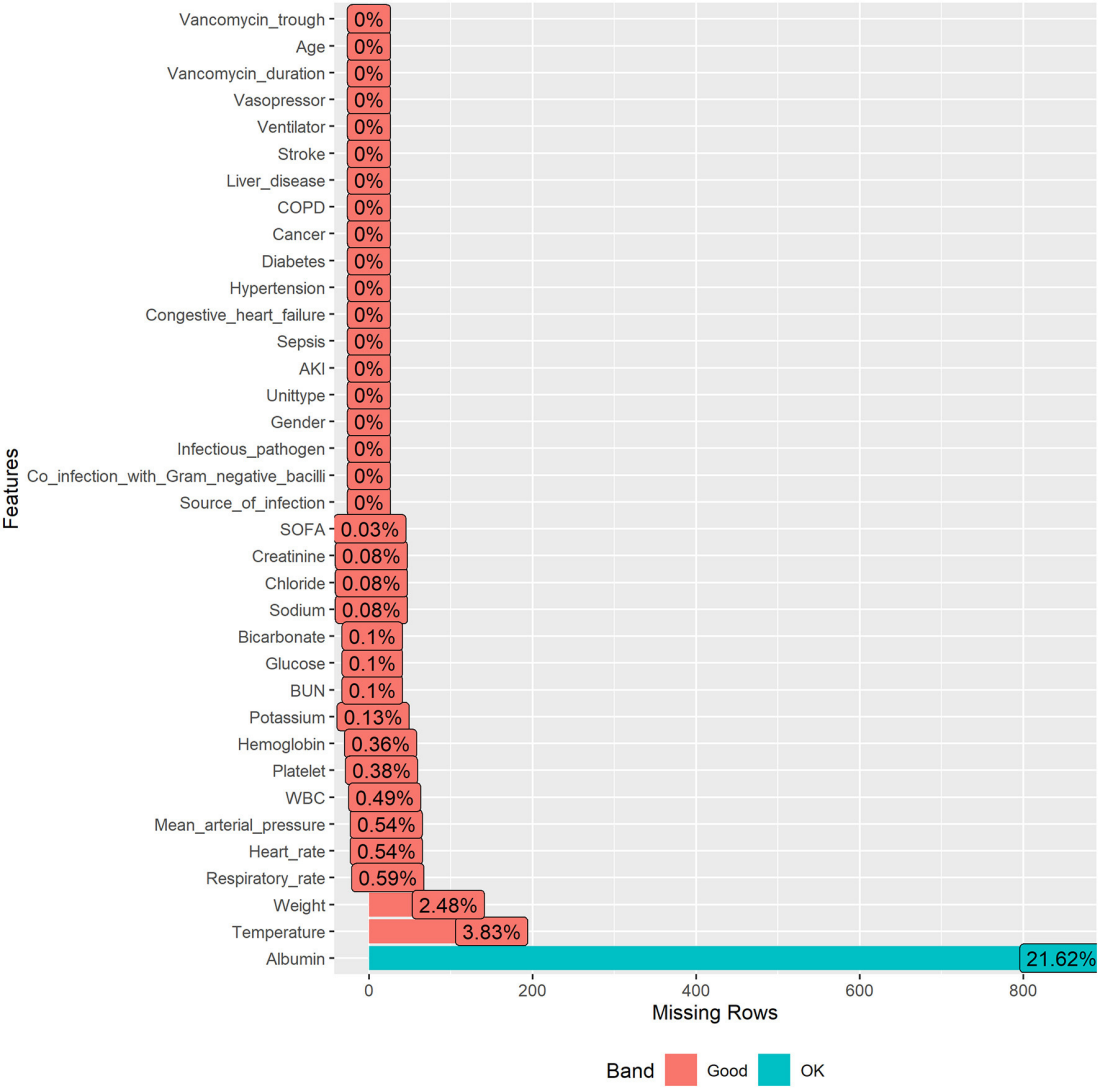
Specific missing rates for variables.

The missing rates for each variable were presented in Figure 2, and because the R package involved in this study allowed for missing values, no further processing of missing values was performed to ensure that they were real data frompatients.

Classification data were expressed as percentages and frequencies, and continuous data were expressed as mean or median values. Generally, in medical research, most regression models have an important assumption: the independent and dependent variables are linearly related. The linear relationship between FVTC and outcomes were analyzed using restricted cubic splines (RCS) previous to formal data analysis. If the value at *P* > 0.05 represented a linear relationship, the following multivariate logistic regression analysis was used to analyze the connection between patient's FVTC and the occurrence of AKI. And multivariate Cox proportional hazard model was used to determine the effect of FVTC on ICU mortality and hospital mortality. Moreover, patients were divided into four groups in the light of the FVTC value: group1 ≤ 10 mg/L, 10 <group 2 ≤ 15 mg/L, 15 <group 3 ≤ 20 mg/L, group4 >20 mg/L (2, 26). The effect of FVTC as a categorical variable on the outcomes was further explored.

Logistic regression and Cox proportional hazards regression models were used to calculate the odd ratios/hazard ratios (ORs/HRs) and their corresponding 95% confidence intervals (CIs). Model I was not adjusted, whereas model II was adjusted for age, weight, gender, SOFA score, the type of patient's first admission to ICU, ventilator use, vasopressor use, comorbidities, source of infection, infectious pathogen, co-infection with Gram-negative bacilli, duration of vancomycin use, and vital signs first monitored within 24 h of entering ICU and the first laboratory test result after entering ICU. Prior to multivariate analysis, variance inflation factors (VIFs) were used to assess multicollinearity between variables.

Subgroup analyses were performed for age (<65 or ≥65 years), gender (male or female), SOFA score (<6 or ≥6), unit type (MICU/SICU or others), ventilator (no or yes) and vasopressor (no or yes) use, sepsis (no or yes), source of infection (hematogenous, lung, abdomen, tissue/bone, others/unclear), infectious pathogen (MRSA, staphylococcus, streptococcus, enterococcus, others/unclear), and co-infection with Gram-negative bacilli (no or yes).

R (version 4.1.0) software were used for statistical analyses. P <0.05 was considered statistically significant.

## Results

### Baseline Results

The study eventually included 3,917 vancomycin-treated patients from the MIMIC-IV database, of whom 2,805 developed AKI and 1,112 did not (Table 1). Patients in the AKI group were older than those in the non-AKI group [65.00 (54.00, 76.00) vs. 59.00 (48.00, 71.00)]; the proportions of male patients in the two groups were 58.6 and 58.8%, respectively; the SOFA score of patients in the AKI group was higher than that in the non-AKI group [3.00 (1.00, 5.00) vs. 1.00 (0.00, 4.00)]. The length of hospital stay and ICU stay in the AKI group were significantly longer than those in the non-AKI group [7.76 (4.70, 13.14) vs. 4.70 (3.11, 7.91) and 16.00 (10.00, 25.00) vs. 15.00 (9.00, 25.00), respectively]. Among comorbidities, sepsis accounted for the highest proportion, at 94.2% in the AKI group and 85.0% in the non-AKI group. The most frequent source of infection was from the lungs, with 44.0 and 38.8% in the two groups, respectively. The remaining characteristics are shown in Table 1.

**Table 1:** Baseline characteristics of the study population.

	**AKI**	**Non-AKI**	***p*-value**
*N*	2,805	1,112	
Age (year)	65.00 (54.00, 76.00)	59.00 (48.00, 71.00)	<0.001
Weight(kg)	80.50 (67.50, 98.20)	71.80 (60.77, 85.02)	<0.001
Gender (%)			0.952
Male	1,645 (58.6)	654 (58.8)	
Female	1,160 (41.4)	458 (41.2)	
SOFA	3.00 (1.00, 5.00)	1.00 (0.00, 4.00)	<0.001
Unit type (%)			0.018
MICU/SICU	2,223 (79.3)	919 (82.6)	
Others	582 (20.7)	193 (17.4)	
Length of stay in ICU (day)	7.76 (4.70, 13.14)	4.70 (3.11, 7.91)	<0.001
Length of stay in hospital (day)	16.00 (10.00, 25.00)	15.00 (9.00, 25.00)	0.014
Ventilator (%)			<0.001
No	275 (9.8)	313 (28.1)	
Yes	2,530 (90.2)	799 (71.9)	
Vasopressor, (%)			<0.001
No	1,206 (43.0)	745 (67.0)	
Yes	1,599 (57.0)	367 (33.0)	
**Comorbidities**‘			
Sepsis (%)			<0.001
No	162 (5.8)	167 (15.0)	
Yes	2,643 (94.2)	945 (85.0)	
Congestive heart failure (%)			<0.001
No	1,878 (67.0)	871 (78.3)	
Yes	927 (33.0)	241 (21.7)	
Hypertension (%)			<0.001
No	1,451 (51.7)	656 (59.0)	
Yes	1,354 (48.3)	456 (41.0)	
Diabetes (%)			<0.001
No	1,971 (70.3)	859 (77.2)	
Yes	834 (29.7)	253 (22.8)	
Cancer (%)			0.319
No	2,359 (84.1)	920 (82.7)	
Yes	446 (15.9)	192 (17.3)	
COPD (%)			<0.001
No	1,924 (68.6)	833 (74.9)	
Yes	881 (31.4)	279 (25.1)	
Liver disease (%)			<0.001
No	2,586 (92.2)	1,063 (95.6)	
Yes	219 (7.8)	49 (4.4)	
Stroke (%)			0.043
No	2,343 (83.5)	898 (80.8)	
Yes	462 (16.5)	214 (19.2)	
Source of infection (%)			0.224
Hematogenous	305 (10.9)	179 (16.1)	
Lung	1,233 (44.0)	432 (38.8)	
Abdomen	232 (8.3)	53 (4.8)	
Tissue/bone	890 (31.7)	394 (35.4)	
Others/unclear	145 (5.2)	54 (4.9)	
Infectious pathogen (%)			0.172
MRSA	253 (9.0)	81 (7.3)	
Staphylococcus	230 (8.2)	107 (9.6)	
Streptococcus	99 (3.5)	49 (4.4)	
Enterococcus	260 (9.3)	105 (9.4)	
others/unclear	1,963 (70.0)	770 (69.2)	
Co-infection with Gram-negative bacilli
No	2,280 (81.3)	890 (80.0)	0.395
Yes	525 (18.7)	222 (20.0)	
Vital signs
Heart rate (min-1)	90.84 (78.30, 103.11)	92.51 (80.83, 103.81)	0.026
Mean arterial pressure (mmhg)	74.48 (69.29, 81.27)	76.56 (69.76, 85.77)	<0.001
Temperature (°C)	36.99 (36.67, 37.42)	37.09 (36.78, 37.49)	<0.001
Respiratory rate (min-1)	20.57 (17.77, 23.92)	20.38 (17.50, 23.91)	0.430
Laboratory tests
WBC (K/uL)	12.50 (8.50, 18.20)	12.00 (8.07, 16.90)	0.009
Hemoglobin (g/dl)	10.20 (8.90, 12.00)	10.30 (8.90, 11.90)	0.787
Platelet (K/uL)	207.00 (137.00, 293.00)	207.00 (138.00, 288.25)	0.952
Sodium (mmol/L)	138.00 (135.00, 141.00)	138.00 (135.00, 141.00)	0.249
Potassium (mmol/L)	4.10 (3.70, 4.50)	4.00 (3.60, 4.40)	<0.001
Chloride (mmol/L)	104.00 (100.00, 108.00)	104.00 (100.00, 108.00)	0.919
Creatinine (mg/dl)	1.00 (0.70, 1.40)	0.90 (0.60, 1.30)	<0.001
BUN (mg/dl)	21.00 (14.00, 34.00)	17.00 (11.00, 29.00)	<0.001
Glucose (mg/dl)	132.00 (107.00, 174.00)	125.00 (104.00, 160.00)	<0.001
Bicarbonate (mmol/L)	23.00 (19.00, 26.00)	23.00 (20.00, 25.00)	0.429
Albumin (mg/dl)	2.70 (2.30, 3.20)	2.90 (2.50, 3.30)	<0.001
Vancomycin trough (mg/L)	16.40 (11.30, 21.80)	14.00 (8.97, 19.10)	<0.001
Vancomycin duration (h)	147.00 (81.00, 255.00)	97.00 (68.00, 169.25)	<0.001
Group			<0.001
≤ 10 (mg/L)	540 (19.3)	339 (30.5)	
>10, ≤ 15 (mg/L)	683 (24.3)	284 (25.5)	
>15, ≤ 20 (mg/L)	688 (24.5)	242 (21.8)	
>20 (mg/L)	894 (31.9)	247 (22.2)	

### Restricted Cubic Splines

**Figure 3:**
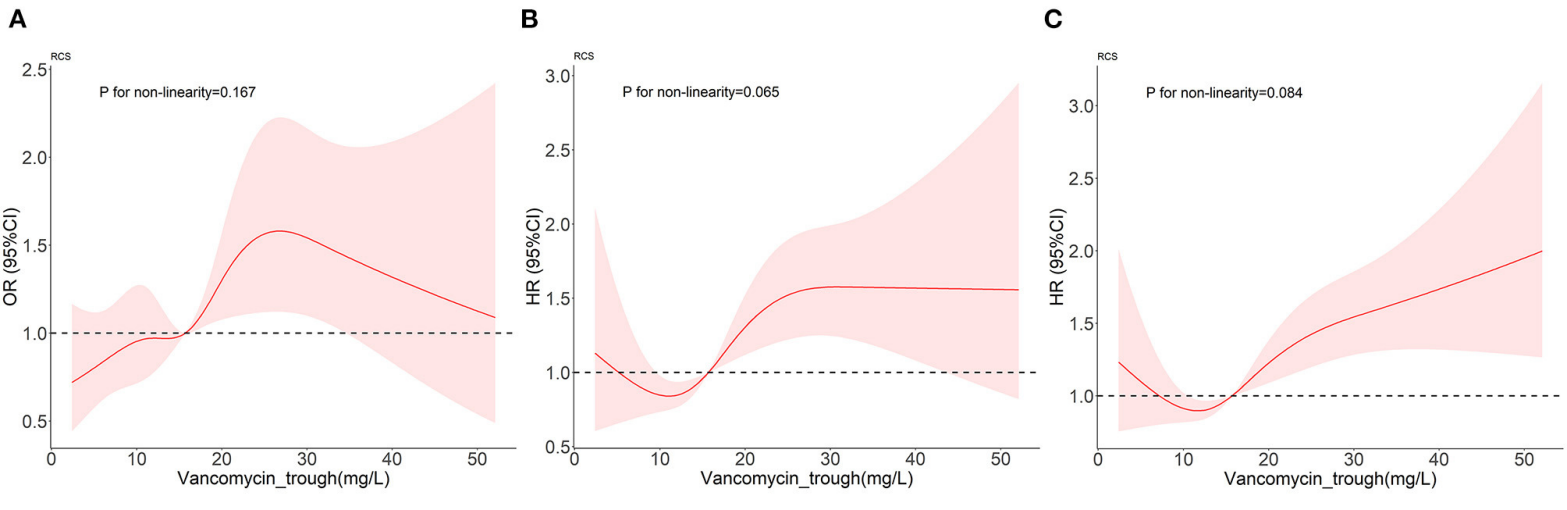
Dose-response relationships between vancomycin trough concentration and the outcomes. **(A–C)** represent the occurrence of AKI, ICU mortality, and in-hospital mortality.

First of all, the RCS in Figure 3 proved the linear relationship between FVTC and the occurrence of AKI as well as between FVTC and ICU mortality and hospital mortality (P for non-linearity >0.05).

### Univariate and Multivariate Analyses

The logistic regression/COX model showed that FVTC, whether as a continuous or a categorical variable, was positively correlated with the occurrence of AKI, ICU mortality, and in-hospital mortality when unadjusted. The results of VIFs prior to multivariate analysis were listed in Supplementary Table S1, with VIFs between 1 and 5 for each variable, illustrating the absence of multicollinearity problems (27). After adjusted by age, weight, gender, SOFA score, type of patient's first admission to ICU, ventilator use, vasopressor use, comorbidities, source of infection, infectious pathogen, co-infection with Gram-negative bacilli, duration of vancomycin use, vital signs, and the laboratory test result, FVTC was still a risk factor with the occurrence of AKI (OR: 1.02; 95% CI:1.01–1.04), ICU mortality (HR:1.02; 95% CI:1.01–1.03), and in-hospital mortality (HR:1.02; 95% CI:1.01–1.03), which means that for every unit increase in FVTC, the risk of AKI, ICU and in-hospital mortality increases by 1.02 times. When FVTC was treated as a categorical variable, group 3 and group 4 had a significant relationship on the occurrence of AKI [group3 (OR:1.36; 95% CI: 1.02–1.81); group 4 (OR: 1.76; 95% CI: 1.32–2.35)]and ICU mortality [group3 (HR: 1.47; 95% CI: 1.03–2.09); group4 (HR: 1.87; 95% CI: 1.33–2.62)], compared to group 1, while group 4 had a significant effect on in-hospital mortality (HR: 1.48; 95% CI: 1.15–1.91). From another point of view, in comparison with group 1, groups 3 and 4 can increase the risk of AKI in patients by 1.36 and 1.76 times, and can increase the risk of ICU mortality by 1.47 and 1.87 times, while group 4 can increase the risk of in-hospital mortality by 1.48 times (Table 2).

**Table 2:** Analysis of the associations between outcomes and vancomycin.

	**Continuous regression model**		**Categorical regression model**					
			**Group1**	**Group2**		**Group3**		**Group4**	
	**OR/HR (95% CI)**	***p*-value**	**OR/HR (95% CI)**	**OR/HR (95% CI)**	***p*-value**	**OR/HR (95% CI)**	***p*-value**	**OR/HR (95% CI)**	***p*-value**
**Primary outcome**
**AKI**
Unadjusted	1.03 (1.02,1.04)	<0.001	Reference	1.51 (1.24,1.83)	<0.001	1.78 (1.46,2.18)	<0.001	2.27 (1.87,2.77)	<0.001
Adjusted	1.02 (1.01,1.04)	0.001	Reference	1.16 (0.88,1.53)	0.300	1.36 (1.02,1.81)	0.037	1.76 (1.32,2.35)	<0.001
**Second outcome**
**ICU mortality**
Unadjusted	1.02 (1.02,1.03)	<0.001	Reference	1.37 (1.02,1.83)	0.037	1.80 (1.35,2.38)	<0.001	1.97 (1.51,2.57)	<0.001
Adjusted	1.02 (1.01,1.03)	<0.001	Reference	1.33 (0.93,1.92)	0.127	1.47 (1.03,2.09)	0.032	1.87 (1.33,2.62)	<0.001
**In-hospital mortality**
Unadjusted	1.03 (1.02,1.03)	<0.001	Reference	1.23 (0.98,1.55)	0.080	1.65 (1.32,2.07)	<0.001	1.89 (1.53,2.33)	<0.001
Adjusted	1.02 (1.01,1.03)	<0.001	Reference	1.01 (0.76,1.33)	0.958	1.22 (0.94,1.60)	0.134	1.48 (1.15,1.91)	0.003

### Subgroup Analyses

In the subgroup analysis between continuous variables of FVTC and the primary outcome AKI, they lacked an interaction (Table 3), suggesting a consistent association between patient populations with different characteristics and AKI risk (28).

**Table 3:** Subgroup analysis of the associations between the occurs of AKI and vancomycin trough concentration (continuous variable).

	**OR (95% CI)**	***p*-value**	***p*-interaction**
Age			0.973
<65 (*n =* 2,021)	1.02 (1.00,1.04)	0.036	
≥65 (*n =* 1,896)	1.04 (1.02,1.06)	<0.001	
SOFA			0.942
<6 (*n =* 3,235)	1.02 (1.01,1.04)	0.015	
≥6 (*n =* 681)	1.02 (0.98,1.06)	0.277	
Gender			0.117
Male (*n =* 2,299)	1.02 (1.01,1.04)	0.042	
Female (*n =* 1,618)	1.03 (1.01,1.06)	0.003	
Unit type			0.392
MICU/SICU (*n =* 3,142)	1.02 (1.01,1.03)	0.005	
Others (*n =* 775)	1.04 (1.01,1.08)	0.025	
Ventilator			0.301
No (*n =* 588)	1.03 (1.01,1.08)	0.009	
Yes (*n =* 3,329)	1.02 (1.01,1.04)	0.006	
Vasopressor			0.957
No (*n =* 1,951)	1.02 (1.01,1.04)	0.010	
Yes (*n =* 1,966)	1.02 (1.00,1.05)	0.035	
Sepsis			0.788
No (*n =* 329)	0.98 (0.92,1.05)	0.542	
Yes (*n =* 3,588)	1.02 (1.01,1.04)	<0.001	
Source of infection			NA
Hematogenous (*n =* 484)	1.01 (0.76,1.56)	0.463	
Lung (*n =* 1,665)	1.03 (1.01,1.05)	0.008	
Abdomen (*n =* 285)	1.02 (0.95,1.11)	0.527	
Tissue/bone (*n =* 1,284)	1.03 (1.01,1.05)	0.014	
Others/unclear (*n =* 199)	NA		
Infectious pathogen			0.253
MRSA (*n =* 334)	1.01 (0.96,1.06)	0.662	
Staphylococcus (*n =* 337)	1.03 (0.98,1.10)	0.284	
Streptococcus (*n =* 148)	1.04 (0.94,1.17)	0.445	
Enterococcus (*n =* 365)	1.01 (0.96,1.06)	0.701	
Others/unclear (*n =* 2,733)	1.03 (1.01,1.05)	<0.001	
Co-infection with Gram-negative bacilli			0.785
No (*n =* 3,170)	1.02 (1.01,1.04)	0.002	
Yes (*n =* 747)	1.02 (0.99,1.05)	0.110	

## Discussion

Vancomycin is widely used, and scholars should focus on the evaluation of its efficacy and adverse reactions. A total of 3,917 critically ill patients treated with vancomycin in MIMIC-IV database were included in this study, of whom 2,805 developed AKI, with an incidence of 71.6%, which was significantly higher than that in other studies. This is due to the complex disease basis, multiple drug combinations and other risk factors of patients in intensive care environment. FVTC was used to study the effect on the incidence of AKI and mortality. After multivariate logistic regression, the risk of AKI increased 1.02 times with 1 unit increase in VTC. Meanwhile, patients with FVTC between 15 and 20 mg/L and >20 mg/L would have 1.36- and 1.76-times higher risk of AKI than those in the control group (<10 g/mL), respectively. Similar trends were observed in the analysis of ICU and in-hospital mortality risk.

Although the mechanisms of vancomycin and nephrotoxicity have not been fully elucidated yet, the following mechanisms have been studied. First, in the dose-dependent mechanism, when vancomycin is excreted by the kidneys, renal tubular epithelial cells can be exposed to the drug for a long time. With increasing concentration in urine, the concentration of the drug continues to increase, and crystals containing drugs and their metabolites directly damage the renal tubules or form a cast obstruction, leading to kidney damage (29). Second, in the dose-independent mechanism, vancomycin can directly increase the oxygen consumption of renal tubular epithelial cells, activate the oxidative stress response, and induce mitochondrial damage, inflammatory response, and other cell apoptosis (30); these phenomena can cause renal tubular ischemia and acute tubular injury (31). In the past, maintaining vancomycin trough concentrations at “15-20 mg/L” was widely used in clinical practice, but an increasing number of published studies have reported a strong relationship between this range and vancomycin-induced nephrotoxicity (32, 33). The present study also reached a consistent conclusion.

It has been supported that maintaining vancomycin at a higher minimum concentration of ≥15 mg/L in the treatment of severe infections can improve the clearance of infectious bacteria and thus improve the prognosis of patients (34). However, our analysis of the secondary outcomes showed that FVTC, whether as a continuous or categorical variable, is a risk factor for ICU mortality and in-hospital mortality. After multiple COX regression analyses, the risk of death in ICU and hospital increased by 1.02 times as the VTC increased by 1 unit. When the patient's FVTC was between 15 and 20 mg/L, it could increase the risk of ICU mortality by 1.47 times, while at the same time, when the FVTC was higher than 20 mg/L, the risk of ICU and in-hospital mortality increased by 1.87 and 1.48 times, respectively. When the trough concentration of vancomycin is too high, it will not benefit the infected patients but may also induce nephrotoxicity and other drug-related side effects and increase the mortality of patients. Therefore, in clinical applications, after ensuring that vancomycin achieves effective treatment outcome, a lower blood concentration can reduce the occurrence of AKI, which is more beneficial to patients. When vancomycin causes kidney damage in patients, it will cause water and electrolyte disorders and abnormal blood coagulation in the body. In critically ill patients, even mild, reversible AKI can increase the risk of death (35). In 2020, the latest version of the guidelines revised the previous treatment principles and pointed out that maintaining the VTC of severely infected patients at 15–20 mg/L as a medication guideline is no longer recommended to avoid the occurrence of adverse reactions (36).

At present, the situation of clinical anti-infective treatment is becoming more and more serious, and the infection of severe patients is often severe and urgent (37). It is recommended that clinicians should also take into account the renal toxicity after drug application when covering possible pathogens in empirical treatment to prevent the occurrence or aggravation of renal impairment in patients (38). For severe patients treated with vancomycin, the treatment plan of vancomycin should be optimized, the FVTC and renal function indexes of patients can be closely observed, the risk factors of vancomycin-related acute kidney injury should be avoided as much as possible, which not only can early detection of renal damage, to avoid irreversible renal damage, but also beneficial to reduce the risk of death of patients.

## Advantages and Limitations

This study used the large public database MIMI-IV to verify the relationship between FVTC and AKI and mortality in critically ill patients. The large sample size provided a benefit to our study. In addition, we proved the connection between FVTC and the outcome from continuous and categorical variables; this finding is more convincing and improves the clinical application value of FVTC. However, this study still has certain limitations. First, because this study was retrospective, some confounding bias was inevitable, so some factors that could potentially affect the patients' renal function like exposure to radioactive contrast dye or drugs were not adjusted for in the model. In addition, this study did not directly analyze the impact of the combination of a specific antibiotic on outcomes. In future studies, further exploration with real-world patient data should be conducted to contribute to the vancomycin dosing regimen.

## Conclusions

FVTC is associated with the occurrence of AKI and increased ICU and in-hospital mortality in critically ill patients. Therefore, in clinical practice, patients in intensive care settings receiving vancomycin should be closely monitored for FVTC to prevent drug-related nephrotoxicity and reduce patient mortality.

## Data Availability Statement

Publicly available datasets were analyzed in this study. This data can be found here: The data were available on the MIMIC-IV website at https://mimic-iv.mit.edu/.

## Ethics Statement

All procedures performed in studies involving human participants were in accordance with the Ethical Standards of the Institutional and National Research Committee and with the 1964 Helsinki declaration and its later amendments or comparable Ethical Standards.

## Author Contributions

LZL and LMZ created the study protocol, performed the statistical analyses, and wrote the first manuscript draft. SJL conceived the study and critically revised the manuscript. FSX assisted with the study design and performed data collection. LL assisted with data collection and manuscript editing. SNL assisted the analysis and explain of statistical methods. HYY assisted with manuscript revision and data confirmation. JL contributed to data interpretation and manuscript revision. All authors read and approved the final manuscript.

## Funding

This work was supported by the National Natural Science Foundation of China (Nos. 82072232 and 81871585), the Natural Science Foundation of Guangdong Province (No. 2018A030313058), Technology and Innovation Commission of Guangzhou Science, China (No. 201804010308). This study was supported by Guangdong Provincial Key Laboratory of Traditional Chinese Medicine Informatization (2021B1212040007).

## Conflict of Interest

The authors declare that the research was conducted in the absence of any commercial or financial relationships that could be construed as a potential conflict of interest.

## Publisher's Note

All claims expressed in this article are solely those of the authors and do not necessarily represent those of their affiliated organizations, or those of the publisher, the editors and the reviewers. Any product that may be evaluated in this article, or claim that may be made by its manufacturer, is not guaranteed or endorsed by the publisher.
